# BRCA 1/2-Mutation Related and Sporadic Breast and Ovarian Cancers: More Alike than Different

**DOI:** 10.3389/fonc.2014.00019

**Published:** 2014-02-27

**Authors:** Melissa Burgess, Shannon Puhalla

**Affiliations:** ^1^Department of Medical Oncology, University of Pittsburgh Medical Center, University of Pittsburgh Cancer Institute, Pittsburgh, PA, USA

**Keywords:** *BRCA 1/2*-mutations, breast cancer, ovarian cancer, BRCAness, PARP inhibitor, reversion mutations

## Abstract

No longer is histology solely predictive of cancer treatment and outcome. There is an increasing influence of tumor genomic characteristics on therapeutic options. Both breast and ovarian cancers are at higher risk of development in patients with *BRCA 1/2*-germline mutations. Recent data from The Cancer Genome Atlas and others have shown a number of genomic similarities between triple negative breast cancers (TNBCs) and ovarian cancers. Recently, poly (ADP-ribose) polymerase (PARP) inhibitors have shown promising activity in hereditary *BRCA 1/2*-mutated and sporadic breast and ovarian cancers. In this review, we will summarize the current literature regarding the genomic and phenotypic similarities between *BRCA 1/2*-mutation related cancers, sporadic TNBCs, and sporadic ovarian cancers. We will also review Phase I, II, and III data using PARP inhibitors for these malignancies and compare and contrast the results with respect to histology.

## Introduction

BRCA1 and 2 proteins play integral functions in DNA homologous recombination repair (HRR). In normal cells, the HRR pathway is activated in response to DNA double-stranded breaks (1). In *BRCA 1/2*-deficient cells, HRR is faulty secondary to loss of *BRCA* function, and therefore, other more error-prone DNA repair pathways are activated. These less perfect mechanisms are felt to be accountable, in part, for carcinogenesis. Similarly, tumors with defective HRR mechanisms are more susceptible to the direct DNA damaging effects of chemotherapy.

Homologous recombination repair dysfunction can be exploited as a therapeutic strategy by the use of poly (ADP-ribose) polymerase (PARP) inhibitors, which inhibit PARP proteins, most commonly PARP1 and 2. As part of the base excision repair (BER) pathway, PARP1 attaches long polymers of ADP-ribose on itself, so that, XRCC1 and other repair proteins have the ability to rapidly locate single-stranded DNA breaks (2–4). Newer evidence reveals that the exact role of PARP1 in the BER pathway is perhaps more indirect and not yet clearly defined (5). Recent studies have also shown that PARP1 is more versatile, and has been implicated in other DNA repair pathways, such as the non-homologous end-joining (NHEJ) repair pathway (6, 7).

Several mechanisms by which PARP inhibition in HRR-deficient cells lead to cell death have been investigated. Most notably, the concept of synthetic lethality explains combinatory lethal effects of BER and HR repair dysfunction, whereas alone, HR or BER pathway disruptions are not lethal to the tumor cell (8). Additionally, other potential mechanisms have been explored including trapping of inhibited PARP1 at sites of DNA damage preventing other repair proteins access, failure to initiate HRR by PARP-dependent BRCA1 recruitment, and activation of the error-prone NHEJ repair pathway leading to genomic instability and subsequent cell death (9). Knowledge of PARP activity has led to effective treatment strategies for *BRCA 1/2*-germline mutation related tumors.

## *BRCA 1/2*-Mutated Ovarian and Breast Cancer

*BRCA 1/2*-mutation related ovarian and breast cancers account for 5–10% of all female ovarian and breast cancers (10, 11). Ovarian cancers in the setting of *BRCA 1/2*-germline mutations can present with more aggressive, high-grade histologies, but are frequently responsive to chemotherapy, particularly platinum-based regimens, leading to an improved 5 years survival (12). The chemotherapy-sensitive mechanism is felt to be related to the intimate relationship between BRCA 1/2 proteins and defective HRR, as discussed above. Recent studies have demonstrated that women with *BRCA*-related ovarian cancers fare much better than sporadic ovarian cancers (13–16). A study, published by the National Israeli Study of Ovarian Cancer, showed women with *BRCA* mutations had a median survival of 55.7 months compared to 37.9 months in sporadic ovarian cancers (*p* = 0.002) (15). This may be in part explained by the standard use of carboplatin-based therapies for ovarian malignancies as the DNA damage induced by the platinum should be more efficacious in the DNA repair-deficient *BRCA*-related tumors.

Contrary to the more convincing outcomes in *BRCA 1/2*-related ovarian cancers, the outcomes of *BRCA* mutation-related breast cancers are less clear. Women with *BRCA1* mutations typically develop breast cancer at an earlier age than *BRCA2*-related and sporadic breast cancers. *BRCA1*-related breast cancers tend to also be higher grade, hormone receptor-negative, and HER-2-negative, or “triple negative” (17), and also frequently express a basal phenotype (18–26). Patients with *BRCA*-mutated breast cancers generally respond to therapy as well as sporadic cancers; however, the risk of second ipsilateral or contralateral primaries may be as high as 3–5% per year, compared to 0.5–1% per year risk, seen in sporadic breast cancers (17). In contrast to ovarian cancer, platinum chemotherapy is not standardly administered to patients with breast cancer. The use of platinum agents has been evaluated in a small series which have demonstrated high efficacy in breast cancer in particular in the setting of a *BRCA* mutation. Silver et al. evaluated the use of neoadjuvant platinum-containing chemotherapy in patients with triple negative breast cancer (TNBC) (*N* = 28), and found those more likely to be platinum-sensitive were those with low *BRCA1* gene expression (27). Likewise, in *BRCA*-mutated breast cancer patients who received cisplatin in the neoadjuvant setting showed a high rate of pathologic complete response (pCR) in a small series. Ten of 12 patients achieved pCR (83%). When non-platinum-containing regimens were used, the pCR rate was 14% (28). These studies highlight the rationale to further explore the use of platinum-containing regimens, specifically for patients with TNBC and *BRCA* mutations.

## BRCAness: Sporadic Triple Negative Breast Cancers

Triple negative breast cancers account for ~20% of all breast cancers and are associated with an aggressive clinical picture (20, 25, 29). Due to lack of hormone receptor or HER-2 expression, and no other known target for tailored therapy, the only current treatment option is chemotherapy. Over 80% of hereditary *BRCA1*-mutated cancers are TNBCs. Several studies have investigated a potential role for *BRCA1* inactivation in sporadic TNBC given the similar clinical outcomes and histological characteristics among these cancers and hereditary *BRCA1*-mutated breast cancers. Breast cancers developing in patients with *BRCA1* mutations, in addition to frequently being triple negative, also often express basal markers (18–22, 25, 26). Gene microarray expression profiling has shown considerable similarities between *BRCA1*-mutated tumors and basal tumors (25). This shared phenotype has been termed “BRCAness” (26). What is unknown is whether the basal phenotype is a result of the *BRCA* loss or if the *BRCA* loss results in the basal phenotype (6).

Recently, Lehmann and colleagues delved further into the characterization of TNBC. They performed an analysis of gene expression profiles of 587 TNBC cases and identified six separate subtypes of TNBC. These six subtypes were: basal-like 1 (BL1), basal-like 2 (BL2), immunomodulatory (IM), mesenchymal (M), mesenchymal stem-like (MSL), and luminal androgen receptor (LAR) subtype. Additional analysis of TNBC cell lines, representative of each of these identified subsets, revealed differential responses to various therapeutic agents. Both the BL1 and BL2 groups showed increased gene expression involved in DNA damage response, and showed higher response to cisplatin (30). In a follow-up study, Masuda et al. presented neoadjuvant chemotherapy response data in each of the aforementioned TNBC subtypes (31). In 130 TNBC patients, who received standard anthracycline- and taxane-based chemotherapy, the BL1 subtype achieved a pCR most frequently (52%). In contrast, the pCR in the BL2 subtype was 0%. The molecular differences in BL1 and BL2 may explain these differential responses. Specifically, the BL1 subtype involves the cell cycle, DNA replication reactome, and the *BRCA* pathway, among others, whereas the BL2 subtype involves growth factor, glycolysis, and gluconeogenesis pathways. This work demonstrates that even within “basal-like breast cancer (BLBC),” there may be a great deal of heterogeneity.

Telli and colleagues recently presented a study evaluating gemcitabine, carboplatin, and iniparib, a compound initially believed to have PARP inhibitory effects, in the neoadjuvant treatment of triple negative and *BRCA*-mutated breast cancer (32). This study demonstrated a pCR of 36% overall, with a pCR in *BRCA 1/2*-mutation carriers of 47%. Furthermore, patients who were both triple negative and had a *BRCA 1/2*-mutation, had a pCR of 56%. Although only 10 patients were classified as BL1 or BL2, there were an equal number of responders and non-responders to the neoadjuvant platinum regimen. It is also notable that only one patient classified as basal-like had a known *BRCA* mutation, whereas, there were *BRCA*-mutated tumors that were classified as IM, M, MSL, and unspecified (32). Although basal-like TNBC has become nearly synonymous with BRCAness, this study found that the basal-like subtype of TNBC was neither particularly responsive to the treatment combination, nor had a higher number of *BRCA*-germline mutations. In this study, the homologous recombination deficiency (HRD) score appeared to be more predictive of platinum response, as compared to TNBC intrinsic subtyping (30). The HRD assay has been developed to evaluate for loss of heterozygosity (LOH), which has been shown to be predictive of response to platinum in *BRCA*-related and sporadic cancers (33). While, this data is hypothesis-generating and thought-provoking, larger, prospective studies will be needed before any formal conclusions can be drawn.

In sporadic basal tumors, there are data that show reduced BRCA1 mRNA expression. It is felt that epigenetic modification of the *BRCA* gene, such as promoter hypermethylation, is responsible for this (34–36). Interestingly, no tumors showed both *BRCA1* mutation and *BRCA1* promoter methylation suggesting that these events are mutually exclusive in The Cancer Genome Atlas (TCGA) research network data (37). The association between *BRCA1*-mutated and BLBCs provides an important rationale to include this frequently encountered patient population in studies geared toward manipulation of the characteristic faulty DNA repair mechanisms in *BRCA1*-mutated tumors. As we move into an era where genomic analyses of tumors is becoming the norm, it will be important to link the genome, methylome, and proteome to clinical characteristics and outcomes.

## BRCAness: Sporadic High-Grade Serous Ovarian Cancers

Similarly, there are many commonalities among *BRCA 1/2*-mutated cancers and sporadic epithelial ovarian cancers (EOCs). Although only 5–10% of ovarian cancers are directly attributable to a germline mutation in *BRCA1* or *2*, there is a growing body of evidence to suggest that additional mechanisms of *BRCA* dysfunction are involved in the pathogenesis of ovarian cancer (26, 38, 39). One study demonstrated alterations of *BRCA1* and/or *2* in up to 82% of examined ovarian cancers (*n* = 92) (40). Methylation of the *BRCA1* promoter has been demonstrated in up to 14% of sporadic breast and up to 30% of sporadic ovarian cancers (26, 35, 41–46). LOH has been described in ovarian tumors and may have multiple possible mechanisms leading to malignancy including co-existing LOH of *BRCA1* and *p53*, and hypermethylation acting in a synergistic fashion (33, 47–51). In contrast, *BRCA2* methylation has not been found to be a significant contributor (39, 52). Identifying and manipulating these *BRCA*-like deficiencies in DNA repair in sporadic ovarian cancers is of great importance and provides rationale for including these patients in clinical trials designed for *BRCA*-related malignancies.

Another important mechanism of BRCAness in ovarian cancers is the presence of somatic mutations in *BRCA1* and *2* (53). Hennessy and colleagues performed *BRCA1/2* sequencing on 235 unselected ovarian cancers and found that 19% of the sample had detectable mutations in *BRCA1* (*N* = 31) or *BRCA2* (*N* = 13). In the 28 samples, where germline DNA was also available, 42.9% of the *BRCA1* mutations and 28.6% of the *BRCA2* mutations were somatic. Of interest, somatic *BRCA 1/2*-mutations in breast cancer appear to be less frequent. In the TCGA BLBC cohort, about 20% had either germline (*N* = 12) or somatic (*N* = 8) *BRCA 1/2*-mutations. Another study evaluated 77 TNBC samples and only one harbored a somatic *BRCA* mutation (54). This potentially explains the seemingly higher activity of single agent PARP inhibitors, discussed later, in sporadic ovarian cancer as compared to sporadic TNBC.

## Genomic Similarities: Basal-Like Breast Cancer and High-Grade Serous Ovarian Cancers

The Cancer Genome Atlas network recently published findings again demonstrating the four distinct molecular signatures in breast cancer from diverse genetic and epigenetic alterations: luminal A, luminal B, basal-like, and HER-2 enriched subtypes (55). Strikingly, BLBCs were notably different than the other three subtypes based on comprehensive analyses using multiple platforms. As expected, these cancers also often (80%) lacked expression of ER, PR, and HER-2 identifying as TNBCs. Specifically, most BLBCs showed a high frequency of *TP53* deleterious mutations (80%), as well as, loss of *RB1* and *BRCA1*. *PIK3CA* mutations (~9%) were also a common feature of BLBC. Analyses also highlighted increased *MYC* activation as a BLBC characteristic.

The BLBC mutation spectrum reported in the TCGA was similar to that identified in previously described serous ovarian cancers (56) and BLBC were more similar to serous ovarian carcinomas than to other subtypes of breast cancer. One gene, in particular, *TP53*, had a >10% mutation frequency in both basal-like breast and serous ovarian cancers. As well, both tumors when compared to luminal showed increased *BRCA1* inactivation, *RB1* loss, cyclin E1 amplification, high expression of *AKT3*, and *MYC* amplification. These molecular commonalities strongly suggest shared driving events in tumorigenesis, and similarly, show support for shared treatment strategies for TNBCs and high-grade serous ovarian cancers. Of note, *p53* mutations have been described to have high frequency in *BRCA* mutation-related cancers as well (57, 58).

## PARP Inhibitors: Preclinical Era

Bryant et al. and Farmer et al. demonstrated synthetic lethality in *BRCA2*-deficient cells with the use of two different PARP inhibitors (59, 60). PARP inhibitors have also shown efficacy preclinically in cells lacking other HRR proteins, such as *RAD51*, *ATR*, *ATM*, *CHK1*, and *FANCA* or *FANCC* (61). These studies have given basis for clinical trials in both *BRCA*-deficient cancer populations, as well as, those with malignancies sharing qualities of BRCAness or HRR-deficiency, such as basal-like or TNBC and serous ovarian cancer.

## PARP Inhibitors in Clinical Trials

### *BRCA 1/2*-mutation studies

The first published Phase I study evaluating PARP inhibitors in the clinic used olaparib (AZD2281) enrolling patients with varying malignancies (Tables 1 and 2) (62). An expansion cohort of *BRCA*-positive ovarian, breast, and prostate cancer patients was enrolled at the recommended Phase II dose of 400 mg twice daily. Nearly half of the evaluable patients had an objective response (19 patients, 47%). Results from this pivotal study showed olaparib was generally well tolerated. From here, two Phase II proof-of-concept trials (ICEBERG 1 and 2) (Tables 1 and 2) confirmed activity in both *BRCA*-mutated ovarian and breast cancers, with olaparib at 400 mg twice daily [ORR 11/33 (33%) and 11/27 (41%), respectively], with low overall toxicities (63, 64).

Olaparib was also evaluated in patients with sporadic cancers displaying a presumed BRCAness phenotype. Gelmon et al. performed a non-randomized Phase II trial using olaparib in heavily treated high-grade serous or undifferentiated ovarian carcinomas and TNBCs (65) (Tables 1–4). Stratified by *BRCA* mutation status, both *BRCA*-mutated and *BRCA*-wild type ovarian carcinoma patients showed response to olaparib. In contrast, neither *BRCA*-mutated nor sporadic breast cancer patients demonstrated significant response to olaparib. Potential explanations for these mixed results include that not all TNBCs have a *BRCA*-like phenotype, so there may have been some heterogeneity to this population (30).

**Table 1:** **Selected PARP inhibitor trials in *BRCA 1/2*-mutated (*BRCA*^mut^) breast cancers**.

Trial	Study population	PARP inhibitor	Comparison therapy	Clinical responses^a^
Phase I	Advanced *BRCA*ut^mut^ tumors (*N* = 39, of which 8 BC)	BMN 673	None	*BRCA*ut^mut^ BC
De Bono et al. (71)				ORR: 2/6
NCT01286987	
Phase I	Advanced solid tumors/hematologic malignancies (*N* = 100, of which 12 BC, including 4 *BRCA*ut^mut^)	Niraparib	None	*BRCA*ut^mut^ BC
Sandhu et al. (68)				PR: 2/4
NCT00749502	
Phase I	Advanced solid tumors	Olaparib	None	*BRCA*ut^mut^ BC
Fong et al. (62)	*N* = 60, of which 9 BC, including 3 with *BRCA*ut^mut^			CR: 1/3
NCT00516373				SD: 1/3
Phase II	Recur, advanced *BRCA*ut^mut^ OC (*N* = 17)/BC (*N* = 10), or *BRCA*t^wt^ HGS and/or undifferentiated OC (*N* = 47)/TNBC (*N* = 16)	Olaparib	None	*BRCA*ut^mut^ BC
Gelmon et al. (65)				CR + PR: 0/8
NCT00679783				SD: 5/8
Phase II	*BRCA*ut^mut^ solid tumors (BC, *N* = 62, OC, *N* = 193)	Olaparib	None	*BRCA*ut^mut^ BC
Kaufman et al. (89)				CR: 0/62
NCT01078662				PR: 8/62
				SD: 29/62
				PFS rate: 29% for 6 months
				OS rate: 44.7% for 12 months
Phase II	*BRCA*ut^mut^ advanced BC (*N* = 27)	Olaparib	None	ORR: 11/27
Tutt et al. (64)				CR: 1/27
NCT00494234				PR: 10/27
ICEBERG 1				PFS: 5.7 months
Phase I	Met or unresect *BRCA*ut^mut^ BC and EOC (*N* = 45, of which 8 BC)	Olaparib + carboplatin	None	*BRCA*ut^mut^ BC
Lee et al. (72)				CR: 1/8
NCT00647062, NCT01445418				PR: 6/8
				SD: 1/8
Phase I	Advanced solid tumors [*N* = 87, including BC (26%) and OC (7%), of which 12 *BRCA*ut^mut^]	Olaparib + carboplatin ± paclitaxel	None	*BRCA*ut^mut^
van der Noll et al. (90)				CR: 17%^b^
NCT00516724				PR: 33%^b^
Phase I	Recur or advanced EOC/TNBC	Olaparib + cediranib (angiogenesis inhibitor)	None	*BRCA*ut^mut^ BC
Liu et al. (82)	*N* = 28, of which 3 *BRCA*ut^mut^ BC			ORR: 0/3
NCT01116648	
Phase I/II Kristeleit et al. (69) NCT01482715	Advanced solid tumors and relapsed PSens *BRCA*ut^mut^ OC	Rucaparib	None	*BRCA*ut^mut^ BC PR: 1/17
	*N* = 29, of which 17 BC and 7 OC, including *BRCA*ut^mut^ tumors			SD: 10/29 (of which 4 were BC, also 7/10 were *BRCA*ut^mut^)^b^
Phase I	Advanced *BRCA*ut^mut^ solid tumors (*N* = 38, of which 12 BC), or *BRCA*t^wt^ BLBC or OC	Veliparib	None	*BRCA*ut^mut^ BC
Huggins-Puhalla et al. (91)				PR: 1/12
NCT00892736				SD: 10/38^b^
Phase I Ramaswamy et al. (92) NCT01251874	Met or unresect *BRCA*ut^mut^ BC, or *BRCA*t^wt^ TNBC and other BCs	Veliparib + carboplatin	None	*BRCA*ut^mut^ BC PR: 2/6
	*N* = 38, of which 6 *BRCA*ut^mut^ and 7 FAef^def^			SD: 4/6
				PR: 8/38^b^
				SD: 17/38^b^
Phase I	Met or unresect *BRCA*ut^mut^ BC	Veliparib + carboplatin	None	CR: 3/26^b^
Somlo et al. (93)	*N* = 28			PR: 9/26
NCT01149083				SD: 7/26
				PFS: 7.8 months
Phase I Rodler et al. (94) NCT01104259	Met *BRCA*ut^mut^ BC or recur and/or met *BRCA*t^wt^ TNBC	Veliparib + cisplatin and vinorelbine	None	*BRCA*ut^mut^ BC PR: 3/5
	*N* = 18, of which 5 *BRCA1/2*ut^mut^			
				PR: 6/11^b^
				SD: 5/11^b^
Phase I	Met BC	Veliparib + cyclophosphamide and doxorubicin	None	PR: 2/11 (both *BRCA2*ut^mut^)
Tan et al. (95)	*N* = 11, of which 3 *BRCA2*ut^mut^			SD: 6/11 (of which 1 *BRCA2*ut^mut^)
NCT00740805	
Phase II	Met *BRCA*ut^mut^ BC (expansion cohort, *N* = 24)	Veliparib + temozolomide	None	CR: 1/24
Isakoff et al. (96)				PR: 2/24
NCT01009788				SD: 7/24

*^a^Data include only patients with measurable disease*.

*^b^Collective data reported*.

*BC, breast cancer; ORR, objective response rate; PR, partial response; CR, complete response; SD, stable disease; recur, recurrent; OC, ovarian cancer; *BRCA*^wt^, *BRCA*-wild type; HGS, high-grade serous; TNBC, triple negative breast cancer; PFS, progression-free survival; OS, overall survival; met, metastatic; unresect, unresectable; EOC, epithelial ovarian cancer; PSens, platinum-sensitive; BLBC, basal-like breast cancer; FA^def^, fanconi anemia pathway deficiency*.

**Table 2:** **Selected PARP inhibitor trials in *BRCA 1/2*-mutated (*BRCA*^mut^) ovarian cancers**.

Trial	Study population	PARP inhibitor	Comparison therapy	Clinical responses^a^
Phase I	Advanced *BRCA*ut^mut^ tumors (*N* = 39, of which 8 BC and 23 OC)	BMN 673	None	*BRCA*ut^mut^ OC
De Bono et al. (71)				ORR: 11/17
NCT01286987	
Phase I	Advanced solid tumors/hematologic malignancies (*N* = 100, of which 49 OC, including 22 *BRCA*ut^mut^)	Niraparib	None	*BRCA*ut^mut^ OC
Sandhu et al. (68)				PR: 8/20
NCT00749502	
Phase I	Advanced solid tumors	Olaparib	None	*BRCA*ut^mut^ OC
Fong et al. (62)	*N* = 60, of which 21 OC, including 16 with *BRCA*ut^mut^			PR: 8/15
NCT00516373				SD: 1/15
Phase II	Recur, advanced *BRCA*ut^mut^ OC (*N* = 17)/BCs (*N* = 10), or *BRCA*t^wt^ HGS and/or undifferentiated OC (*N* = 47)/TNBC (*N* = 16)	Olaparib	None	*BRCA*ut^mut^ OC
Gelmon et al. (65)				CR: 0/17
NCT00679783				PR: 7/17
				SD: 6/17
Phase II	Advanced PRef or PRes *BRCA*ut^mut^ OC	Olaparib	Liposomal doxorubicin	Olaparib 200 mg twice daily
Kaye et al. (66)				PFS: 6.5 months
NCT00628251				ORR: 25%
				Olaparib 400 mg twice daily
				PFS: 8.8 months
				ORR: 31%
				Liposomal doxorubicin:
				PFS: 7.1 months
				ORR: 18%
Phase II	*BRCA*ut^mut^ solid tumors (BC, *N* = 62, OC, *N* = 193)	Olaparib	None	*BRCA*ut^mut^ OC
Kaufman et al. (89)				CR: 6/193
NCT01078662				PR: 54/193
				SD: 78/193
				PFS rate: 54.6% for 6 months
				OS rate: 64.4% for 12 months
Phase II	Advanced *BRCA*ut^mut^ OC	Olaparib	None	ORR: 11/33
Audeh et al. (63)				CR: 2/33
NCT00494442				PR: 9/33
				PFS: 5.8 months
Phase I	Met or unresect *BRCA*ut^mut^ BC and EOC (*N* = 45, of which 37 OC)	Olaparib + carboplatin	None	*BRCA*ut^mut^ OC
Lee et al. (72)				CR: 0/34
NCT00647062, NCT01445418				PR: 15/34
				SD: 14/34
Phase I	Advanced solid tumors *N* = 87, including BC (26%) and OC (7%), of which 12 *BRCA*ut^mut^	Olaparib + carboplatin ± paclitaxel	None	*BRCA*ut^mut^
van der Noll et al. (90)				CR: 17%^b^
NCT00516724				PR: 33%^b^
Phase I	Recur or advanced EOC/TNBC	Olaparib + cediranib (angiogenesis inhibitor)	None	*BRCA*ut^mut^ OC
Liu et al. (82)	*N* = 28, of which 12 *BRCA*ut^mut^ OC			CR: 1/11
NCT01116648				PR: 4/11
Phase I/II Kristeleit et al. (69) NCT01482715	Advanced solid tumors and relapsed PSens *BRCA*ut^mut^ OC	Rucaparib	None	*BRCA*ut^mut^ OC PR: 1/7
	*N* = 29, of which 17 BC and 7 OC, including *BRCA*ut^mut^ tumors			SD: 10/29 (of which 5 were OC, also 7 were *BRCA*ut^mut^)^b^
				CR + PR + SD: 6/7 in OC
Phase I	Advanced *BRCA*ut^mut^ solid tumors (*N* = 38, of which 20 OC), or *BRCA*t^wt^ BLBC or OC	Veliparib	None	*BRCA*ut^mut^ OC
Huggins-Puhalla et al. (91)				PR: 1/20
NCT00892736				SD: 10/38^b^
Phase II Kummar et al. (97) NCT01306032	Refractory progressive *BRCA*ut^mut^ OC or HGS OC	Veliparib (V) + cyclophosphamide (C)	Cyclophosphamide (C)	V + C: PR: 3/36^b^ C: PR: 5/38^b^
		*N* = 36	*N* = 38	
Phase I	Met or unresect solid tumors	Veliparib + carboplatin and gemcitabine	None	CR: 2/59^b^
Bell-McGuinn et al. (98)	*N* = 59, of which 39 OC, 24 of 39 OC *BRCA*ut^mut^			PR: 11/59^b^
NCT01063816				Of 13 responses, 8 *BRCA*ut^mut^ OC, 3 other OC

*^a^Data include only patients with measurable disease*.

*^b^Collective data reported*.

*BC, breast cancer; OC, ovarian cancer; ORR, objective response rate; PR, partial response; SD, stable disease; recur, recurrent; *BRCA*^wt^, *BRCA*-wild type; HGS, high-grade serous; TNBC, triple negative breast cancer; PRef, platinum-refractory; PRes, platinum-resistant; PFS, progression free survival; OS, overall survival; CR, complete response; met, metastatic; unresect, unresectable; EOC, epithelial ovarian cancer; PSens, platinum-sensitive; BLBC, basal-like breast cancer*.

**Table 3:** **Selected PARP inhibitor trials in sporadic breast cancers**.

Trial	Study population	PARP inhibitor	Comparison therapy	Clinical responses^a^
Phase II	Recur, advanced *BRCA*ut^mut^ OC (*N* = 17)/BCs (*N* = 10), or *BRCA*t^wt^ HGS, and/or undifferentiated OC (*N* = 47)/TNBC (*N* = 16)	Olaparib	None	*BRCA*t^wt^ TNBC
Gelmon et al. (65)				CR + PR: 0/15
NCT00679783				SD: 2/15
Phase I	Refractory or recur BC (*N* = 4) and OC	Olaparib + carboplatin	None	BC
Lee et al. (99)				PR: 3/4
NCT01237067				SD: 1/4
Phase I	Advanced solid tumors *N* = 87, including BC (26%) and OC (7%), of which 12 *BRCA*ut^mut^	Olaparib + carboplatin ± paclitaxel	None	ORR: 14/87 (16%)^b^
van der Noll et al. (90)				CR: 5%
NCT00516724				PR: 11%
				SD: 28%
Phase I	Recur or advanced EOC/TNBC	Olaparib + cediranib (angiogenesis inhibitor)	None	BC
Liu et al. (82)	*N* = 28, of which 8 BC			ORR: 0/7
NCT01116648				SD: 2/7
Phase I	Advanced solid tumors	Olaparib + cisplatin	None	CR: 1/54^b^
Balmana et al. (100)	*N* = 54, of which 42 BC			PR: 17/54^b^
NCT00782574				SD: 23/54^b^
Phase I	Met TNBC	Olaparib + paclitaxel	None	PR: 7/19
Dent et al. (76)	*N* = 19			SD: 1/19
NCT00707707	
Phase I	Advanced *BRCA*ut^mut^ solid tumors, or *BRCA*t^wt^ tumors (*N* = 25, of which 21 BLBC)	Veliparib	None	*BRCA*t^wt^ BLBC
Huggins-Puhalla et al. (91)				PR: 1/21
NCT00892736				*BRCA*t^wt^
				SD: 7/25^b^
Phase I	Refractory solid tumors/lymphoma	Veliparib	Cyclophosphamide	PR: 7/35^b^
Kummar et al. (101)	*N* = 35, including BC and OC			SD: 6/35^b^
NCT00810966	
Phase I Ramaswamy et al. (92) NCT01251874	Met or unresect *BRCA*ut^mut^ BC, or *BRCA*t^wt^ TNBC and other BCs	Veliparib + carboplatin	None	PR: 8/38 SD: 17/38
	*N* = 38, of which 6 *BRCA*ut^mut^ and 7 FAef^def^			
				FAef^def^
				PR: 2/7
				SD: 5/7
Phase I	Met or unresect solid tumors	Veliparib + carboplatin and gemcitabine	None	CR: 2/59^b^
Bell-McGuinn et al. (98)	*N* = 59, of which 10 BC			PR: 11/59^b^
NCT01063816				Of 13 responses, 8 *BRCA*ut^mut^ OC, 3 other OC, 2 others
Phase I	Advanced solid tumors including BC	Veliparib + carboplatin and paclitaxel	None	BC
Appleman et al. (102)	*N* = 68, of which 14 BC			CR: 3/14
NCT00535119				PR: 5/14
Phase I	Met or unresect solid tumors, including BC (Q1 week, *N* = 10 TNBC, Q3 week, *N* = 9 TNBC)	Veliparib + carboplatin and paclitaxel	None	TNBC
Puhalla et al. (80) NCT01281150				(Q1 week), CR: 2/10, PR: 3/10, SD: 3/10
				(Q3 week), CR: 3/9, PR: 4/9, SD: 1/9
Phase I Rodler et al. (94) NCT01104259	Met *BRCA*ut^mut^ BC or recur and/or met *BRCA*t^wt^ TNBC	Veliparib + cisplatin and vinorelbine	None	PR: 6/11^b^ SD: 5/11^b^
	*N* = 18, of which 5 *BRCA1/2*ut^mut^			
Phase I Tan et al. (95) NCT00740805	Met BC	Veliparib + cyclophosphamide and doxorubicin	None	PR: 2/11 (both *BRCA2*ut^mut^)
	*N* = 11, of which 3 *BRCA2*ut^mut^			SD: 6/11 (of which 1 *BRCA2*ut^mut^)

*^a^Data include only patients with measurable disease*.

*^b^Collective data reported*.

*recur, recurrent; *BRCA*^mut^, mutated *BRCA*; OC, ovarian cancer; BC, breast cancer; *BRCA*^wt^, *BRCA*-wild type; HGS, high-grade serous; TNBC, triple negative breast cancer; CR, complete response; PR, partial response; SD, stable disease; ORR, objective response rate; EOC, epithelial ovarian cancer; met, metastatic; BLBC, basal-like breast cancer; FA^def^, fanconi anemia pathway deficiency*.

**Table 4:** **Selected PARP inhibitor trials in sporadic ovarian cancers**.

Trial	Study population	PARP inhibitor	Comparison therapy	Clinical responses^a^
Phase II	Recur, advanced *BRCA*ut^mut^ OC (*N* = 17)/BCs (*N* = 10), or *BRCA*t^wt^ HGS and/or undifferentiated OC (*N* = 47)/TNBC (*N* = 16)	Olaparib	None	*BRCA*t^wt^ OC
Gelmon et al. (65)				CR: 0/46
NCT00679783				PR: 11/46
				SD: 18/46
Phase II	Relapsed PSens serous OC after two courses of platinum-based chemotherapy	Olaparib	Placebo	PFS: 8.4 months
Ledermann et al. (81)				OS 29.7 months
NCT00753545				ORR: 12.3%
				ORR + SD: 52.9%
Phase I	Refractory or recur BC (*N* = 4) and OC (*N* = 23)	Olaparib + carboplatin	None	OC
Lee et al. (99)				PR: 8/23
NCT01237067				SD: 11/23
Phase I	Advanced solid tumors *N* = 87, including BC (26%) and OC (7%), of which 12 *BRCA*ut^mut^	Olaparib + carboplatin ± paclitaxel	None	ORR: 14/87 (16%)^b^
van der Noll et al. (90)				CR: 5%
NCT00516724				PR: 11%
				SD: 28%
Phase II	Advanced PSens serous OC	Olaparib + carboplatin, paclitaxel	Carboplatin, paclitaxel alone	PFS: 12.2 months
Oza et al. (103)				ORR: 64%
NCT01081951	
Phase I	Recur or advanced EOC/TNBC	Olaparib + cediranib (angiogenesis inhibitor)	None	OC
Liu et al. (82)	*N* = 28, of which 20 OC			CR: 1/18^b^
NCT01116648				PR: 7/18^b^
				SD: 3/18^b^
Phase I	Advanced solid tumors	Olaparib + cisplatin	None	CR: 1/54^b^
Balmana et al. (100)	*N* = 54, of which 10 OC			PR: 17/54^b^
NCT00782574				SD: 23/54^b^
Phase I	Advanced solid tumors (*N* = 23, of which 6 OC)	Rucaparib + carboplatin	None	OC
Molife et al. (104)				PR: 1/6
NCT01009190				SD: 2/6
Phase I	Advanced *BRCA*ut^mut^ solid tumors, or *BRCA*t^wt^ tumors (*N* = 25, of which 4 OC)	Veliparib	None	*BRCA*t^wt^
Huggins-Puhalla et al. (91)				SD: 7/25^b^
NCT00892736	
Phase I	Refractory solid tumors/lymphoma	Veliparib	Cyclophosphamide	PR: 7/35^b^
Kummar et al. (101)	*N* = 35, including BC and OC			SD: 6/35^b^
NCT00810966	
Phase II Kummar et al. (97) NCT01306032	Refractory progressive *BRCA*ut^mut^ OC or HGS OC	Veliparib (V) + cyclophosphamide (C)	Cyclophosphamide (C)	V + C: PR: 3/36^b^
		*N* = 36	*N* = 38	C: PR: 5/38^b^
Phase I	Met or unresect solid tumors	Veliparib + carboplatin and gemcitabine	None	CR: 2/59^b^
Bell-McGuinn et al. (98)	*N* = 59, of which 39 OC, 24 of 39 *BRCA*ut^mut^			PR: 11/59^b^
NCT01063816				Of 13 responses, 8 *BRCA*ut^mut^ OC, 3 other OC, 2 others

*^a^Data include only patients with measurable disease*.

*^b^Collective data reported*.

*recur, recurrent; *BRCA*^mut^, mutated *BRCA*; OC, ovarian cancer; BC, breast cancer; *BRCA*^wt^, *BRCA*-wild type; HGS, high-grade serous; TNBC, triple negative breast cancer; CR, complete response; PR, partial response; SD, stable disease; PSens, platinum-sensitive; PFS, progression free survival; OS, overall survival; ORR, objective response rate; EOC, epithelial ovarian cancer; BLBC, basal-like breast cancer; met, metastatic; unresect, unresectable*.

In a population of *BRCA*-positive recurrent ovarian cancer patients with a platinum-free interval of ≤12 months, olaparib was compared to pegylated liposomal doxorubicin (PLD) in a randomized Phase II trial (*N* = 97) (66) (Table 2). Progression free survival (PFS) was not statistically significantly different for olaparib 200 or 400 mg twice daily (combined or individually) versus PLD (PFS 6.5 versus 8.8 versus 7.1 months, respectively). Where the PFS and ORR were consistent with prior studies for olaparib at 400 mg twice daily, the efficacy of PLD was higher than expected when compared with previous trials. Toxicity profiles were distinct between olaparib (nausea, vomiting, and fatigue) and PLD (stomatitis and palmar-plantar erythrodysesthesia), and overall, the drugs were well tolerated. Although olaparib did not show an improvement in PFS over chemotherapy, these results show that targeted therapy with a PARP inhibitor is as effective as chemotherapy, with potential for improved tolerability.

Other PARP inhibitors have also been studied in clinical trials including niraparib (MK4827) in both *BRCA*-positive and sporadic tumors. This compound’s mechanism of action includes PARP inhibition via a novel PARP trapping mechanism (67). A Phase I study utilizing niraparib monotherapy was recently published that established a maximum tolerated dose of 300 mg/day (*N* = 100) (68) (Table 1). Dose-limiting toxicities (DLTs) were reported in the first cycle including grade 4 thrombocytopenia at a dose of 400 mg/day. Non-hematologic DLTs included grade 3 fatigue and grade 3 pneumonitis at lower doses (30 and 60 mg/day, respectively). Common treatment-related effects were anemia, nausea, fatigue, thrombocytopenia, anorexia, neutropenia, constipation, and vomiting, but were predominantly grade 1 or 2. There were anti-tumor responses seen in the *BRCA*-mutated breast and ovarian cancer population, and these were recorded at doses >60 mg/day. Results from this study show promise for this newer PARP inhibitor and currently there are multiple Phase III trials recruiting in *BRCA*-positive breast and ovarian, and sporadic ovarian cancer populations (NCT01905592, NCT01847274) (Tables 5 and 6).

**Table 5:** **Ongoing or future PARP inhibitor trials in *BRCA 1/2*-mutated (*BRCA*^mut^) breast and ovarian cancers**.

Trial	Study population	PARP inhibitor	Comparison therapy	ClinicalTrials.gov status
Phase III	Met or unresect *BRCA*ut^mut^ BC	BMN 673	Physician’s choice – capecitabine, eribulin, gemcitabine, or vinorelbine	NCT01945775 Recruiting
Phase III	HER-2 negative met or advanced *BRCA*ut^mut^ BC	Niraparib	Physician’s choice (select from four active comparators)	NCT01905592 (BRAVO) Not yet open for recruitment
Phase III	PSens *BRCA*ut^mut^ or HGS OC w/prior CR and second CR/PR	Niraparib (maintenance)	Placebo	NCT01847274 Recruiting
Phase III	PSens *BRCA*ut^mut^ (stage III or IV) OC in first CR/PR	Olaparib (maintenance)	Placebo	NCT01844986 Not yet open for recruitment
Phase III	Relapsed PSens *BRCA*ut^mut^ OC w/prior CR and second CR/PR	Olaparib (maintenance)	Placebo	NCT01874353 Not yet open for recruitment
Phase II	Met or locally advanced *BRCA*ut^mut^ BC/OC	Rucaparib	None	NCT00664781
				Active, not recruiting
Phase II Miller et al. (105)	*BRCA*ut^mut^ BC or *BRCA*t^wt^ TNBC w/residual disease in adjuvant setting (after NAC/surgery)	Rucaparib + cisplatin	Cisplatin	NCT01074970 Ongoing, not recruiting
Phase I	Met or unresect *BRCA*ut^mut^ BC and OC	Veliparib	None	NCT01853306
				Recruiting
Phase I/II	Relapsed PRes or partially PSens *BRCA*ut^mut^ EOC	Veliparib	None	NCT01472783
				Veli-BRCA
				Recruiting
Phase II	Met or advanced *BRCA*ut^mut^ BC	Veliparib	Placebo and carboplatin, paclitaxel	NCT01506609
Isakoff et al. (106)		Three arms, plus temozolomide, or carboplatin, paclitaxel		Recruiting
Phase II	Advanced or recur *BRCA*ut^mut^ EOC	Veliparib	None	NCT01540565
Coleman et al. (107)				Ongoing, not recruiting
Phase I	*BRCA*ut^mut^ solid tumors (e.g., BC and OC)	Veliparib + oxaliplatin and capecitabine	None	NCT01233505
				Recruiting
Phase I	Met or unresect *BRCA*ut^mut^ BC and OC	Veliparib + temozolomide	None	NCT00526617
				Completed

*met, metastatic; unresect, unresectable; BC, breast cancer, PSen, platinum-sensitive; HGS, high-grade serous; OC, ovarian cancer; CR, complete response; PR, partial response; *BRCA*^wt^, *BRCA*-wild type; TNBC, triple negative breast cancer; NAC, neoadjuvant chemotherapy; PRes, platinum-resistant; EOC, epithelial ovarian cancer; recur, recurrent*.

**Table 6:** **Ongoing or future PARP inhibitor trials in sporadic breast and ovarian cancers**.

Trial	Study population	PARP inhibitor	Comparison therapy	ClinicalTrials.gov status
Phase III	PSens *BRCA*ut^mut^ or HGS OC w/prior CR and second CR/PR	Niraparib (maintenance)	Placebo	NCT01847274
				Recruiting
Phase I	Recur TNBC/HGS OC	Olaparib + BKM120 (PI3 kinase inhibitor)	None	NCT01623349
				Recruiting
Phase I	Met or unresect TNBC/serous EOC	Olaparib + carboplatin	None	NCT01445418
				Recruiting
Phase I/Ib	Relapsed stage III or IV OC	Olaparib + carboplatin and paclitaxel	None	NCT01650376
				Recruiting
Phase II	Relapsed recur PSens high-grade EOC	Rucaparib	None	NCT01891344 (ARIEL2)
				Recruiting
Phase II Miller et al. (105)	*BRCA*ut^mut^ BC or *BRCA*t^wt^ TNBC w/residual disease in adjuvant setting (after NAC/surgery)	Rucaparib + cisplatin	Cisplatin	NCT01074970 Ongoing, not recruiting
Phase I	Recur or residual EOC/met TNBC	Veliparib	Pegylated liposomal doxorubicin	NCT01145430
Pothuri et al. (108)				Recruiting
Phase I	Recur met or locally advanced unresect solid tumors (e.g., BC/OCs) with organ dysfunction	Veliparib	Carboplatin and paclitaxel	NCT01366144 Recruiting
Phase I	Recur OC	Veliparib	None	NCT01459380
	Two arms + doxorubicin, carboplatin, and bevacizumab			Recruiting
Phase I	Node-positive BC with incomplete response to NAC	Veliparib	Radiation therapy	NCT01618357
				Recruiting
Phase I	Recur stage IV EOC	Veliparib + intraperitoneal floxuridine (FUDR)	None	NCT01749397
				Recruiting
Phase I	Newly diagnosed stage II–IV optimally or suboptimally debulked OC	Veliparib + paclitaxel, carboplatin, bevacizumab	None	NCT00989651 Recruiting
		Two parallel arms		
Phase II	Stage IIA, IIIA–C TNBC	Veliparib + paclitaxel + carboplatin, followed by doxorubicin, cyclophosphamide (neoadjuvant)	Paclitaxel, carboplatin, followed by doxorubicin, cyclophosphamide	NCT01818063
Avery et al. (109)				Recruiting
Phase II	Recur HGS OC	Veliparib + temozolomide	Pegylated liposomal doxorubicin	NCT01113957
				Completed
Phase I/II	Recurrent, relapsed PRes or part PSens OC	Veliparib + topotecan	None	NCT01690598
				Recruiting
Phase II	Recur advanced non-PSens OC	Veliparib + topotecan	None	NCT01012817
				Recruiting

*PSen, platinum-sensitive; *BRCA*^mut^, *BRCA 1/2*-mutated; HGS, high-grade serous; OC, ovarian cancer; CR, complete response; PR, partial response; recur, recurrent; TNBC, triple negative breast cancer; met, metastatic; unresect, unresectable; EOC, epithelial ovarian cancer; BC, breast cancer; *BRCA*^wt^, *BRCA*-wild type; NAC, neoadjuvant chemotherapy; PRes, platinum-resistant*.

Rucaparib (CO-338/AG-014699, also previously PF-01367338) was recently evaluated in Phase I and II studies in advanced solid tumors, including *BRCA*-positive breast and ovarian cancers. The PARP inhibitor as monotherapy and in combinations with cytotoxic chemotherapy is under investigation. In a standard dose-escalation fashion, a Phase I/II study (Tables 1 and 2) is currently evaluating rucaparib monotherapy in advanced solid tumors (*N* = 29) including ovarian/primary peritoneal (*N* = 7) and breast (*N* = 17) cancer patients (69). Thus far, no DLTs at 360 mg twice daily (study not yet complete) have been reported. To date, two PRs were seen in one *BRCA*-positive ovarian cancer, and one *BRCA*-positive breast cancer patient at 300 mg daily dosing during the sixth week of therapy. Ten additional patients (ovarian *N* = 5, breast *N* = 4, and colorectal *N* = 1) have experienced stable disease (SD) at >12 weeks so far; seven of which are *BRCA*-positive. Overall the disease control rate (PR + SD > 12 weeks) for ovarian cancer patients is 86% (6/7). Further results are anticipated from this study. These promising results to date have supported ARIEL2, a Phase II study of rucaparib in platinum-sensitive, relapsed, high-grade epithelial ovarian, fallopian tube, or primary peritoneal cancer patients, which is currently recruiting patients (Table 6).

BMN 673, a novel, highly potent PARP 1/2 inhibitor, demonstrated high efficacy in preclinical studies (70). BMN 673 elicits DNA repair biomarkers at much lower concentrations [PARP1 half maximal inhibitory concentration (IC_50_) <1 nmol/L] than earlier generation PARP inhibitors, i.e., olaparib, veliparib, and rucaparib. Its anti-tumor activity has been tested *in vitro* and in xenograft cancer models, as monotherapy and in combination. Anti-tumor activity was seen in *BRCA1*, *BRCA2*, and *PTEN* deficient cells with a 20 to more than 200-fold greater potency than existing PARP 1/2 inhibitors. Synergism was also seen when BMN 673 was combined with temozolomide, SN38, or platinum drugs. Thus far, BMN 673 has been the most specific PARP inhibitor in its class.

The first in-human Phase I, clinical trial using BMN 673 in solid tumor patients was recently presented at ASCO 2013 (71) (Tables 1 and 2). Patients with advanced solid tumors defective in DNA repair, including *BRCA*-mutated breast (*N* = 6), and ovarian (*N* = 17) cancer patients, were eligible for the stage II expansion phase at the maximum tolerated dose of 1000 mcg daily. In total, 39 patients with advanced solid tumors were enrolled, including those tumors with deleterious *BRCA* mutations. Thrombocytopenia was dose-limiting and occurred in three patients at doses 900 or 1100 mcg daily. Most potential treatment-related adverse events (AEs) were grade 1/2 and included fatigue, nausea, flatulence, anemia, neutropenia, thrombocytopenia, and alopecia. Objective responses were seen in 11/17 *BRCA*-mutated ovarian/primary peritoneal cancer patients and 2/6 *BRCA*-mutated breast cancer patients. Based on these encouraging results, the recommended dose, 1000 mcg daily, will be studied in a Phase III trial in *BRCA*-carrier metastatic or locally advanced breast cancer patients (NCT01945775) (Table 5).

In addition to the single agent studies described above, PARP inhibitors have been combined with chemotherapy in *BRCA* mutation-related malignancies. Lee et al. in a Phase I/Ib study, utilized olaparib, in combination with carboplatin, in a standard dose-escalation study design in *BRCA 1/2*-mutated breast and ovarian cancers (*N* = 45) (72) (Tables 1 and 2). The recommended Phase II dose was 400 mg twice daily for 14 days with carboplatin AUC 5. As noted in several other trials utilizing olaparib, and other PARP inhibitors, myelosuppression was frequently present with grade 3/4 AEs (neutropenia 42%), as well as, thrombocytopenia (20%), anemia (13%), carboplatin-hypersensitivity (9%), and fatigue (7%). Responses included one CR in a breast cancer patient that was durable (duration of 17 months), and a PR in 15/34 (44%) ovarian cancer (duration 3–28+ months) and 6/8 breast cancer (duration 5–24+ months) patients. Prolonged SD was seen in 14/34 (41%) ovarian cancer patients for as long as 25 months and for 11 months in a breast cancer patient. Remarkably, the overall clinical benefit rate was 100% in breast cancer patients and 85% in ovarian cancer patients. A summary of Phase I–III studies utilizing PARP inhibitors in *BRCA* 1/2-mutated breast and ovarian cancers can be found in Tables 1 and 2.

### Sporadic breast and ovarian cancer trials

The earliest trials reported for sporadic TNBCs evaluated iniparib (BSI-201) in combination with gemcitabine and carboplatin. The Phase II trials showed promising anti-tumor activity, prolonged median progression-free survival, and median overall survival (OS) with minimal overall toxicity (73). Disappointingly, the results were not significant in the Phase III trial (74). There are a number of potential explanations for the lack of efficacy seen in the Phase III study, including the heterogeneity within the subtypes of TNBC. Importantly, it was discovered that iniparib was actually not a PARP inhibitor, at physiologic concentrations. Rather, iniparib was shown to cause telomere-centric DNA damage (75).

There are also a number of reported and ongoing studies with “true” PARP inhibitors in sporadic TNBCs, although, only a few studies that have been published in final format. A Phase I/II study of mention explored the use of olaparib in combination with paclitaxel in the first or second-line setting for metastatic TNBC patients (*N* = 19) (76) (Table 3). Notably, patients were treated with olaparib 200 mg daily with paclitaxel 90 mg/m^2^ weekly for 3 of 4 weeks and 15 of the patients had had previous taxane-based therapy. Thirty-seven percent of patients had a PR, although, there were significant dose modifications due to the greater than expected rate of neutropenia, even despite use of growth factor support. While taxanes are proven agents in TNBC (77–79), this class is not typically thought to be a potentiating agent for PARP inhibitors. Most studies have used a platinum agent for potentiation, exploiting the DNA damage/dysfunctional DNA repair pathways concept. Perhaps utilizing two agents that are active in different parts of the cell cycle would potentially target more tumor cells, overall, including those in different phases of growth. Additionally, the utility of PARP inhibitor/taxane-based combination may have potentially overcome taxane resistance. There are ongoing studies with platinum and taxane combinations with a PARP inhibitor. Early looks at efficacy are promising (80).

Similarly in ovarian cancer, there have been a number of studies evaluating PARP inhibitors with chemotherapy, including in the maintenance setting. Ledermann et al. studied olaparib in the maintenance setting after second CR in platinum-sensitive recurrent serous ovarian cancer patients. This was a Phase II, randomized, double-blinded, placebo-controlled trial (*N* = 265) (81) (Table 4). Median PFS was statistically significant between the groups, 8.4 versus 4.8 months, in the olaparib and placebo arms, respectively (*p* < 0.001). OS was not significantly different (29.7 versus 29.9 months in the olaparib and placebo groups, respectively). Further studies are needed to identify a population of patients that may experience greater clinical benefit, such as those with *BRCA 1/2*-mutations or those with a BRCAness phenotype.

Combination therapies with PARP inhibitors have also been investigated in sporadic ovarian and breast cancers, specifically with other novel targeted agents. Cediranib, an anti-angiogenesis agent, was studied with olaparib in recurrent epithelial ovarian or TNBCs (*N* = 28, 20 ovarian and 8 breast) (82) (Tables 1–4). Patients were enrolled to four dose levels and the recommended Phase II dose was cediranib 30 mg daily and olaparib 200 mg twice daily was based on one occurrence of grade 4 neutropenia (≥4 days) and one of grade 4 thrombocytopenia with dosages of cediranib 30 mg daily and olaparib 400 mg twice daily. Seventy-five percent of patients experienced grade 3 or higher toxicities with grade 3 hypertension and fatigue, occurring in 25 and 18% of subjects, respectively. Despite the frequent hematologic and non-hematologic toxicities, the ORR was 44% in the evaluable ovarian cancer population (*N* = 18). Sixty-one percent of ovarian patients had clinical benefit (including those with SD). None of the breast cancer patients experienced clinical response, but two patients had SD for >24 weeks. A summary of Phase I–III studies utilizing PARP inhibitors in sporadic breast and ovarian cancers can be found in Tables 3 and 4.

## Platinum and PARP Inhibitor Resistance

*BRCA 1/2*-deficient cancers are known to be hypersensitive to platinum agents which are thought account for, in part, better overall prognosis for those patients with *BRCA 1/2*-germline mutation-related breast and ovarian cancer. Not all patients respond to platinum, however, and indeed, it is likely that the majority of tumors will eventually become platinum-resistant. Additionally, not all patients with *BRCA 1/2*-germline mutations or those with an expected BRCAness phenotype respond to PARP inhibition. Several mechanisms of resistance to both agents have been hypothesized and are likely to be multifactorial in etiology. Current evidence suggests that secondary mutations occur in the *BRCA1* or *BRCA2* gene restoring the wild type *BRCA 1/2* open reading frame which may provide return of DNA repair through a functional HR pathway. These reversion mutations are thought to lead to platinum resistance, as well as PARP inhibitor resistance (83–87). It is imperative that these secondary mutations are identified to help modulate therapeutic management of these populations. Of interest, PARP inhibitor resistance may, in fact, not affect subsequent therapy response, including subsequent platinum regimens (88).

## Conclusion

Poly (ADP-ribose) polymerase inhibitors have shown promising activity as both monotherapy and in combination with cytotoxic chemotherapy in BRCA 1/2-mutated cancers. More recently, this concept has been implicated in sporadic high-grade serous ovarian cancers and TNBCs. Like platinum agents, PARP inhibitors have been efficacious in this population. Published data from the TCGA network further support this therapeutic strategy by showcasing the genomic similarities between high-grade serous ovarian cancers and TNBCs. It may be worthwhile in the future to study new drug therapies in tandem in these two populations. New strategies are needed to combat tumor resistance mechanisms, such as secondary mutations that revert *BRCA* genes to wild type, to both platinum agents and PARP inhibitors. Future directions for PARP inhibition include when best to use these agents, in what combinations, and precisely, how to define the optimal populations that will get the most benefit.

## Conflict of Interest Statement

The authors declare that the research was conducted in the absence of any commercial or financial relationships that could be construed as a potential conflict of interest.
